# Complete mitochondrial genome of *Aleochara* (*Aleochara*) *curtula* (Goeze, 1777) (Coleoptera: Staphylinidae)

**DOI:** 10.1080/23802359.2023.2167472

**Published:** 2023-01-24

**Authors:** Chan-Jun Lee, Ji-Wook Kim, Jeesoo Yi, Yeon‐Jae Choi, Sangil Kim, Mi-Jeong Jeon, Jong-Seok Park, Sung-Jin Cho

**Affiliations:** a Department of Biological Sciences and Biotechnology, Chungbuk National University, Cheongju, Republic of Korea; b Museum of Comparative Zoology and Department of Organismic and Evolutionary Biology, Harvard University, Cambridge, MA, USA; c School of Biological Sciences, Seoul National University, Seoul, Republic of Korea; d Animal Resources Division, National Institute of Biological Resources, Incheon, Republic of Korea

**Keywords:** Aleocharinae, mitogenome, rove beetle

## Abstract

The mitochondrial genome (mitogenome) of *Aleochara* (*Aleochara*) *curtula* (Goeze, 1777) (Coleoptera: Staphylinidae) is reported. This mitogenome (GenBank accession no. OL675411) is 16,600 bp in size and consists of 13 protein-coding genes (PCGs), 22 transfer RNA genes (tRNAs), and two ribosomal RNA genes (rRNA). Most PCGs use typical mitochondrial stop codon (TAR) except for *cox3*, which uses a single T residue. The A, G, T, and C nucleotide base composition of the mitogenome is 40.61%, 7.66%, 40.34%, and 11.39%, respectively. The phylogenetic analyses recovered the monophyly of Aleocharinae.

Staphylinidae is one of the largest coleopteran families with approximately 63,600 species (Irmler et al. 2018), and the subfamily Aleocharinae Fleming contains over 16,200 described species distributed worldwide (Leschen and Newton 2015). The aleocharine genus *Aleochara* Gravenhorst is recorded by over 400 species in 19 subgenera worldwide and 17 species in five subgenera in Korea (Ahn et al. 2017). Members of this genus are potential biocontrol agents against hygienic pests such as flies. Adults and larvae of the *Aleochara* species are ectoparasitoids of cyclorrhaphous Diptera, laying eggs near the fly larvae so that their offspring can trace the pupae (Klimaszewski 1984). In this study, we sequenced the complete mitochondrial genome of *Aleochara* (*Aleochara*) *curtula* (Goeze, 1777), a rove beetle species reported from regional outbreaks in the Republic of Korea and known to attack vacationers in the region when agitated (Lee 2019).

The specimens of *A. curtula* were collected in Cheongju-si, Chungcheongbuk-do, Republic of Korea (GPS coordinates: 36°37′N 127°27′E), which deposited at the Chungbuk National University Insect Collection (CBNUIC), Cheongju, Republic of Korea (voucher number: CBNU-202005-001; the person in charge of the collection: Jong-Seok Park, jpark16@cbnu.ac.kr). The total genomic DNA was extracted from an adult specimen of *A. curtula* using the MagAttract HMW DNA Kit (QIAGEN Biotech Co., Ltd) according to the manufacturer’s protocol. DNA library was prepared according to Illumina Truseq DNA PCR-free library preparation protocol and sequenced on the NovaSeq6000 sequencer (DNALink, Republic of Korea), using one Illumina lane. A total of 146,923,211 reads and 22,185,404,861 bp were generated. The mitochondrial genome sequence was assembled by GetOrganelle 1.6.4 (Jin et al. 2020) The mitogenome annotation was performed on the MITOS web server (Bernt et al. 2013) and adjusted with Geneious R10 (Biomatters, Auckland, New Zealand).

The total length of the complete mitochondrial genomes of *A. curtula* (GenBank accession no. OL675411) is 16,600 bp, which could be assembled into one circular contig. The A, G, T, and C nucleotide base composition of the mitogenome is 40.61%, 7.66%, 40.34%, and 11.39%, respectively. In general, the PCGs of *A. curtula* have an ATK start codon and a TAR stop codon sequence, with the exception of the *nad1* gene, which uses the TTG start codon and the *cox3* gene, which uses a stop codon with a single T residue.

Maximum likelihood (ML) phylogenetic tree was reconstructed using IQ-TREE V.1.6.9 (Lin et al. 2018; Cai 2021) based on the 13 PCG nucleotide sequence data from our newly sequenced *A. curtula* mitogenome and 18 other species of Staphylinidae, together with one species of Leiodidae as an outgroup from GenBank (Mckenna et al. 2015; Lin et al. 2018; Cai et al. 2019; Kim et al. 2021). Each PCG was separately aligned using the L-INS-i algorithm of MAFFT 7.310 (Katoh and Toh 2008) and concatenated afterward for phylogenetic analysis. The best partitioning scheme and optimal nucleotide substitution models were determined within IQ-TREE: (1) GTR + F + I + G4 for *nad2*, *cox1*, *cox3*, *nad3*, *nad4*, *nad4l* and *cytb*; (2) GTR + F + R3 for *cox2*; (3) TVM + F + I + G4 for *atp8*; (4) TIM + F + I + G4 for *atp6*, *nad5*, *nad6,* and *nad1*. The standard bootstrap analysis was repeated 1,000 times to calculate bootstrap support values. Based on the ML tree reconstructed in this study, *A. curtula* is confirmed to constitute a monophyletic clade with other species of Aleocharinae (Figure 1). Bayesian Inference was conducted by MrBayes 3.2.7 (Ronquist et al. 2012) with the same sequence alignment (Figure 2). Subfamily Aleocharinae is sister to the other ingroup subfamilies, corresponding to BI phylogeny result inferred from the PCGs amino acid dataset (Figure 1 of Song et al. 2021). Although some clades showed different relationships, both ML and BI results exhibited monophyly of each subfamily. It was also confirmed that mitogenomic data are valuable for analyzing phylogenetic relationships among the families of Coleoptera.

**Figure 1.**
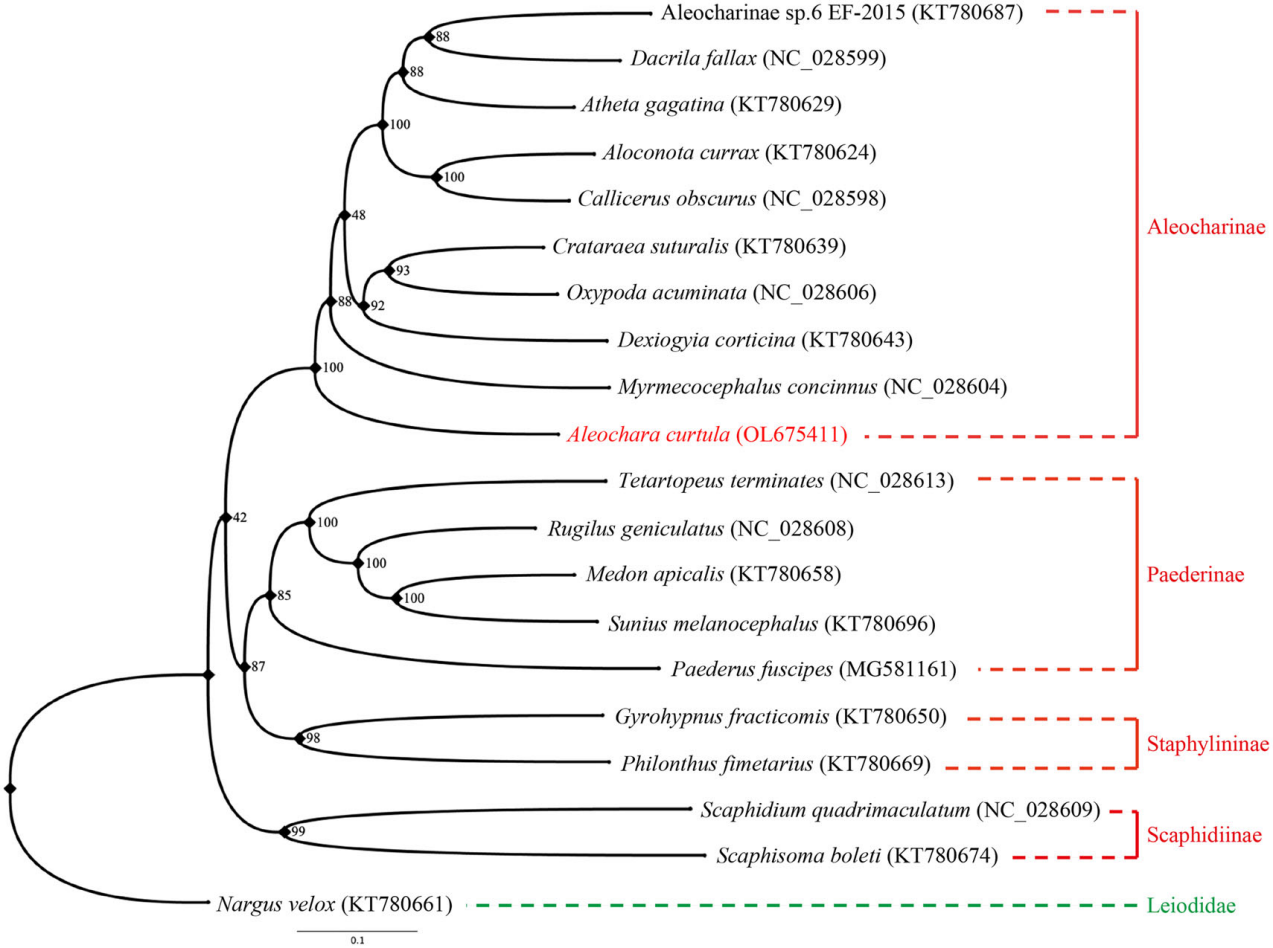
Maximum likelihood phylogeny of Staphylinidae based on the mitogenomic data of *Aleochara* (*Aleochara*) *curtula* and 19 other coleopteran species. Numbers on the nodes indicate bootstrap support values, and tip labels show taxon names with their respective GenBank accession numbers. Our newly sequenced *A. curtula* is marked in red, and the outgroup Leiodidae in green.

**Figure 2.**
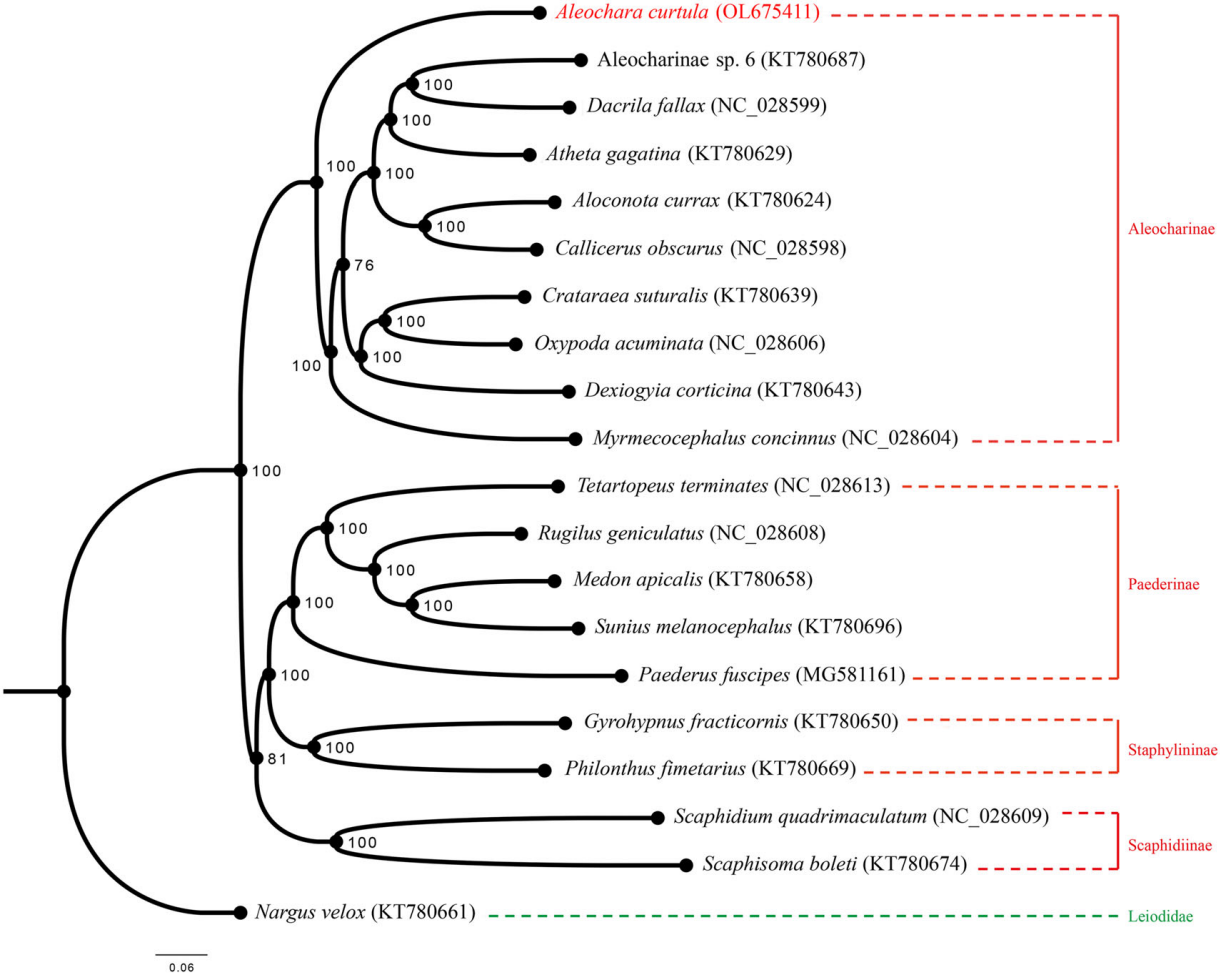
Bayesian Inference of Staphylinidae based on the same alignment data. Numbers on the nodes show the posterior probabilities.

## Data Availability

Mitochondrial genome sequence that supports the findings of this study is openly available in GenBank of NCBI at [https://www.ncbi.nlm.nih.gov] (https://www.ncbi.nlm.nih.gov/) under the accession number OL675411. The associated BioProject, SRA, and Bio-Sample numbers are PRJNA820532, SRX14629784, and SAMN27014688 respectively. FAIRsharing DOI for data repositories is 10.25504/FAIRsharing.99sey6. All samples are stored in CBNUIC (Jong-Seok Park Ph.D, jpark16@cbnu.ac.kr). No applicable regulation or permission is needed for this study, which is related to any ethical issues.
